# Differences in the protein expression levels of Trx2 and Prx3 in the hippocampal CA1 region between adult and aged gerbils following transient global cerebral ischemia

**DOI:** 10.3892/mmr.2015.3760

**Published:** 2015-05-08

**Authors:** CHOONG HYUN LEE, JOON HA PARK, JEONG-HWI CHO, JI HYEON AHN, EUN JOO BAE, MOO-HO WON

**Affiliations:** 1 Department of Pharmacy, College of Pharmacy, Dankook University, Cheonan, South Chungcheong 330-714, Republic of Korea; 2 Department of Neurobiology, School of Medicine, Kangwon National University, Chuncheon, Gangwon 200-701, Republic of Korea; 3 Department of Pediatrics, Chuncheon Sacred Heart Hospital, College of Medicine, Hallym University, Chuncheon, Gangwon 200-702, Republic of Korea

**Keywords:** thioredoxin 2, peroxiredoxin 3, transient global cerebral ischemia, hippocampal CA1 region, stratum pyramidale, aging

## Abstract

The thioredoxin (Trx) and peroxiredoxin (Prx) redox system is associated with neuronal damage and neuroprotective effects via the regulation of oxidative stress in brain ischemia. In the present study, ischemia-induced changes in the protein expression levels of Trx2 and Prx3 in the stratum pyramidale (SP) of the hippocampal CA1 region were investigated in adult and aged gerbils, subjected to 5 min of transient global cerebral ischemia, using immunohistochemistry and western blot analysis. In the adult ischemia-group, minimal Trx2 immunoreactivity was detected in the SP 2 days after ischemia-reperfusion. In the aged animals, the Trx2 immunoreactivity in the sham-group was marginally lower compared with that in the adult sham-group. In the aged ischemia-group, Trx2 immunoreactivity in the SP was significantly higher 1, 2 and 4 days post-ischemia, compared with that in the adult ischemia-group and, in the 5 days post-ischemia group, Trx2 immunoreactivity was significantly decreased in the SP. Prx3 immunoreactivity in the SP of the adult ischemia-group was significantly decreased from 4 days after ischemia-reperfusion. In the aged animals, Prx3 immunoreactivity in the sham-group was also marginally lower compared with that in the adult sham-group. Prx3 immunoreactivity in the aged ischemia-group was also significantly higher 1, 2 and 4 days post-ischemia, compared with the adult ischemia-group; however, the Prx3 immunoreactivity was significantly decreased 5 days post-ischemia. The western blot analyses revealed that the pattern of changes in the protein levels of Trx2 and Prx3 in the adult and aged hippocampal CA1 region following ischemic damage were similar to the results obtained in the immunohistochemical data. These findings indicated that cerebral ischemia lead to different protein expression levels of Trx2 and Prx3 in the hippocampal CA1 region between adult and aged gerbils, and these differences may be associated with more delayed neuronal death in the aged gerbil hippocampus following transient global cerebral ischemia.

## Introduction

Transient global cerebral ischemia leads to selective neuronal damage via triggering a complex series of biochemical events in certain regions of the brain, including the hippocampus and neocortex (1,2). The hippocampal CA1 region, in particular, is well known as the most vulnerable region (3–5). Neuronal death in the hippocampal CA1 region occurs a few days following transient ischemic insult and is referred to as ‘delayed neuronal death’ (2). It has been demonstrated that cerebral ischemia leads to the production of excessive reactive oxygen species (ROS), although the underlying mechanisms, which are associated with the delayed neuronal death and selective neuronal damage, remain to be fully elucidated (6–8). The cerebral ischemia-induced overproduction of ROS can cause morphological and functional alterations of cells, including the alteration of intracellular Ca^2+^ homeostasis, which has been considered the basis of excitotoxicity injury mechanisms (9–11).

The thioredoxin (Trx) and peroxiredoxin (Prx) redox system is important in cellular function by reducing oxidative stress via the regulation of intracellular ROS levels (12–15). Among the subtypes of Trx and Prx, Trx2 and Prx3 are exclusively expressed in the mitochondrial compartment (16,17), and are involved in the control of the antioxidant defence system, cell survival and apoptosis (18–20). In addition, Trx2 and Prx3 are associated with neuronal damage and neuroprotective effects in the brain in response to neurodegenerative disorders and various insults, including brain ischemia (9,21–24).

In our previous studies, time-dependent changes were reported in the expression levels of Trx2 and Prx3 and the neuroprotective effects of Trx2 and Prx3 in a gerbil model of transient cerebral ischemia (22,25). However, transient cerebral ischemia-induced neuronal damage in the hippocampal CA1 region is also affected by various factors, including the duration of ischemia/reperfusion and the age of the experimental animals (26–28). In addition, the time-dependent changes in the expression levels of Trx2 and Prx3 following cerebral ischemia remain to be fully elucidated in aged animals. In the present study, therefore, ischemia-induced changes in the protein expression levels of Trx2 and Prx3 in the hippocampal CA1 region were compared between adult and aged gerbils following 5 min of transient global cerebral ischemia.

## Materials and methods

### Experimental animals

Male Mongolian gerbils (*Meriones unguiculatus*) were obtained from the Experimental Animal Center, Kangwon National University (Chuncheon, South Korea). The Mongolian gerbils were aged 6 months (body weight, 65–75 g) in the adult group, and 24 months (body weight, 75–85 g) in the aged group. The animals (n=196) were housed in a conventional state under stable temperature (23°C) and humidity (60%) with a 12-h light/12-h dark cycle, and were provided with free access to food and water. The procedures for animal handling and care adhered to guidelines in compliance with the current international laws and policies (Guide for the Care and Use of Laboratory Animals, The National Academies Press, 8th Ed., 2011) (29), and were approved by the Institutional Animal Care and Use Committee at Kangwon National University (Chuncheon, South Korea; approval no. KW-130424-1). All of the experiments were performed in a manner to minimize the number of animals used and the suffering caused by the procedures.

### Induction of transient cerebral ischemia

The animals were anesthetized with a mixture of 2.5% isoflurane (Ilsung Pharmaceuticals, Seoul, Korea) in 33% oxygen and 67% nitrous oxide. The bilateral common carotid arteries were isolated and occluded using non-traumatic aneurysm clips (Yasargil FE 723K; Aesculap, Tuttlingen, Germany). The complete interruption of blood flow was confirmed by observing the central artery in retinae under an opthalmoscope (HEINE K180^®^; Heine Optotechnik, Herrsching, Germany). Following 5 min occlusion, the aneurysm clips were removed from the common carotid arteries. The body (rectal) temperature under free-regulating or normothermic (37±0.5°C) conditions was monitored using a rectal temperature probe (TR-100; Fine Science Tools, Foster City, CA, USA) and maintained using a thermometric blanket prior to, during and following surgery until the animals were completely recovered from anesthesia. Thereafter, the animals were maintained on the thermal incubator (Mirae Medical Industry, Seoul, South Korea) to maintain the body temperature of the animals until the animals were sacrificed. The sham-operated animals were subjected to the same surgical procedures, with the exception that the common carotid arteries were not occluded.

### Tissue processing for histology

For histological analysis, section were prepared from the sham- and ischemia-operated adult and aged gerbils (n=7 at each time point) at designated time-points (1, 2, 4, 5 and 7 days) following reperfusion. The animals were anesthetized with sodium pentobarbital (JW Pharm. Co., Ltd., Korea, 40 mg/kg, i.p) and perfused transcardially with 0.1 M phosphate-buffered saline (PBS; pH 7.4; Sigma-Aldrich, St. Louis, MO, USA) followed by 4% paraformaldehyde (Sigma-Aldrich) in 0.1 M phosphate-buffer (PB; pH 7.4; Sigma-Aldrich). The brains were removed and postfixed in the same fixative for 6 h. The brain tissues were then cryoprotected by infiltration with 30% sucrose (Sigma-Aldrich) overnight. Thereafter, frozen tissues were serially sectioned on a cryostat (Leica Microsystems, GmbH, Wetzlar, Germany) into 30 *μ*m coronal sections, which were then collected into six-well plates containing PBS.

### Staining for neuronal damage

To confirm the delayed neuronal death in the hippocampal CA1 region between the adult and aged gerbils following transient cerebral ischemia, NeuN immunohistochemistry was performed, according to the methods of the previous studies (27,28). In brief, for NeuN immunohistochemistry, the sections were sequentially treated with 0.3% hydrogen peroxide (H_2_O_2_; Sigma-Aldrich) in PBS for 30 min and 10% normal goat serum (Vector Laboratories, Inc., Burlingame, CA, USA) in 0.05 M PBS for 30 min. The sections were then incubated with diluted mouse anti-NeuN, a neuron-specific soluble nuclear antigen (1:1,000; cat. no. MAB377; Millipore, Temecula, CA, USA) overnight at 4°C. Thereafter the tissues were exposed to biotinylated goat anti-mouse immunoglobulin (Ig) G (1:200; Vector Laboratories Inc., Burlingame, CA, USA) and streptavidin peroxidase complex (Vector Laboratories, Inc., Burlingame, CA, USA) for 2 h at room temperature. The sections (6 sections/animal) were visualized by staining with 3,3′-diaminobenzidine (Sigma-Aldrich) in 0.1 M Tris-HCl buffer and mounting on gelatin-coated slides. Following dehydration, the sections were mounted using Canada balsam (Kanto, Tokyo, Japan).

In order to quantitatively analyze NeuN immunoreactivity, digital images of the hippocampal tissues were captured using an AxioM1 light microscope (Carl Zeiss AG, Oberkochen, Germany) equipped with a digital camera (Axiocam; Carl Zeiss AG) connected to a PC monitor. The number of NeuN-immunoreactive neurons were counted in a 250×250 *μ*m square applied approximately at the center of the CA1 region using an image analyzing system (Optimas 6.5; CyberMetrics, Inc., Scottsdale, AZ, USA). The tissue sections were selected at 120 *μ*m intervals, and cell counts were obtained by averaging the counts from each animal.

### Immunohistochemistry for Trx2 and Prx3

To compare the changes of Trx2 and Prx3 in the hippocampal CA1 region between adult and aged gerbils, immunohistochemistry for rabbit anti-Trx2 (1:500; cat. no. LF-PA0024, Ab Frontier, Seoul, Korea) and mouse anti-Prx3 (1:500; cat. no. LF-MA0045; Ab Frontier) was performed, according to the above-mentioned method. In order to establish the specificity of the immunostaining, a negative control was used, with only the secondary antibody and without primary antibody. This negative control resulted in the absence of immunoreactivity in any structures.

A total of six sections with a 120 *μ*m interval per animal were selected to quantitatively analyze the Trx2 and Prx3 immunoreactivity. Digital images of the hippocampal CA1 region were captured using an AxioM1 light microscope (Carl ZeissAG), equipped with a digital camera (Axiocam; Carl Zeiss AG) connected to a PC monitor. According to the methods of our previous study (7), semi-quantification of the immunostaining intensities were evaluated using digital image analysis software (MetaMorph 4.01; Universal Imaging Corporation, Downingtown, PA, USA). The level of immunoreactivity was scaled as −, ±, +, ++ or +++ representing no staining (gray scale value ≥200), weakly positive (gray scale value=150–199), moderate (gray scale value=100–149), marked (gray scale value=50–99), or very marked (gray scale value ≤49), respectively.

### Western blot analysis for Trx2 and Prx3

To examine changes in the protein levels of Trx2 and Prx3 in the hippocampal CA1 region following transient cerebral ischemia, the sham- and ischemia-operated adult and aged animals (n=5 at each time point) were analyzed using western blot analysis in the sham, group and 2 and 5 days following reperfusion. Following sacrifice of the animals and removal of their brains, the brains were serially and transversely cut to a thickness of 400 *μ*m on a vibratome (Leica Microsystems GmbH), and the hippocampal CA1 regions were then dissected using a surgical blade. The tissues were homogenized in 50 mM PBS (pH 7.4) containing 0.1 mM ethylene glycol bis (2-aminoethyl Ether)-N,N,N’,N’ tetraacetic acid (pH 8.0; Sigma-Aldrich), 0.2% Nonidet P-40 (Sigma-Aldrich), 10 mM ethylendiamine tetraacetic acid (pH 8.0; Sigma-Aldrich), 15 mM sodium pyrophosphate (Sigma-Aldrich), 100 mM β-glycerophosphate (Sigma-Aldrich), 50 mM NaF (Sigma-Aldrich), 150 mM NaCl (Sigma-Aldrich), 2 mM sodium orthovanadate (Sigma-Aldrich), 1 mM phenylmethylsulfonyl fluoride and 1 mM dithiothreitol (DTT; Sigma-Aldrich). Following centrifugation at 16,000 × g for 20 min at 4°C, the protein levels were determined in the supernatants using a Micro BCA protein assay kit, with bovine serum albumin as the standard (Pierce Biotechnology, Inc., Rockford, IL, USA). Aliquots containing 20 *μ*g total protein were boiled for 5 min in loading buffer containing 150 mM Tris (pH 6.8), 3 mM DTT, 6% SDS, 0.3% bromophenol blue (Sigma-Aldrich) and 30% glycerol. The aliquots were then loaded onto a 10% polyacrylamide gel. Following electrophoresis, the gels were transferred onto nitrocellulose transfer membranes (Pall Life Sciences, East Hills, NY, USA). To reduce background staining, the membranes were incubated with 5% non-fat dry milk in PBS containing 0.1% Tween 20 (Sigma-Aldrich) for 45 min at room temperature, followed by incubation with rabbit anti-Trx2 (1:1,000; Chemicon International, Temecula, CA, USA) or mouse anti-Prx3 (1:1000; cat. no. LF-PA0024; Ab Frontier), and peroxidase-conjugated donkey anti-rabbit IgG or goat anti-mouse IgG (Santa Cruz Biotechnology, Inc., Dallas, TX, USA) for 2 h at room temperature, and an ECL kit (Pierce Biotechnology, Inc.).

The result of the western blot analyses were scanned, and densitometric analysis for the quantification of the bands was performed using Image 1.46 (National Institutes of Health, Bethesda, MD, USA), which was used to count the relative optical density (ROD). The ratio of the ROD was calibrated as the percentage, with the adult sham-operated group designated as 100%.

### Statistical analysis

The data are expressed as the mean ± standard error of the mean. Differences in the means among the groups were statistically analyzed using one-way analysis of variance with Bonferroni’s multiple comparison post-hoc test in order to elucidate ischemia-associated differences among the experimental groups using SPSS 17.0 software (IBM SPSS, Armonk, NY, USA). P<0.05 was considered to indicate a statistically significant difference.

## Results

### Delayed neuronal death in the hippocampus

Neuronal damage in the hippocampal CA1 of the adult and aged gerbils following transient cerebral ischemia was examined using NeuN immunohistochemistry. In the adult and aged sham-operated gerbils, NeuN-immunoreactive neurons in the stratum pyramidale (SP) of the CA1 region were well observed. At 4 days after ischemia-reperfusion, fewer NeuN-immunoreactive neurons were detected in the SP of the CA1 region in the adult gerbil, due to delayed neuronal death. However, in the aged gerbil, numerous NeuN^+^ neurons were found in the SP of the CA1 region 4 days after ischemia-reperfusion, with delayed neuronal death in the aged group observed 5 days after ischemia-reperfusion (data not shown). This finding was consistent with that of our previous study (27,28).

### Changes in Trx2 immunoreactivity

**Figure 1:**
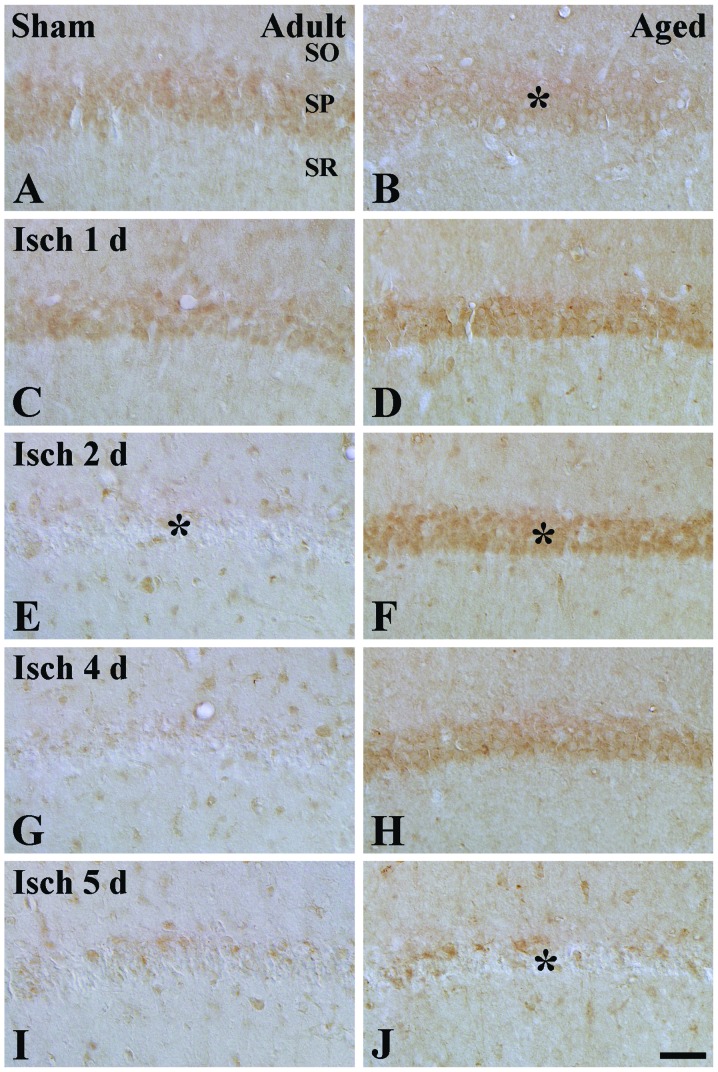
Trx2 immunohistochemistry in the CA1 region of (A and B) sham-operated and (C-J) ischemia-operated (A, C, E, G and I) adult and (B, D, F, H and J) aged gerbils. In the adult ischemia-group, Trx2 immunoreactivity in the SP was decreased 2 days after ischemia-reperfusion (* in E). In the aged sham-group, Trx2 immunoreactivity (* in B) was marginally lower than that in the adult sham-group; however, Trx2 immunoreactivity in the aged ischemia-group was markedly increased (* in F) 1–4 days after ischemia-reperfusion, and significantly decreased (* in J) 5 days after ischemia-reperfusion. Scale Bar=100 *μ*m. SO, stratum oriens; SR, stratum radiatum; SP, stratum pyramidale; Trx, thioredoxin; Prx, peroxiredoxin; sham, no ischemia-reperfusion.

Moderate Trx2 immunoreactivity was detected in the SP of the CA1 region in the adult sham-group, and was marginally higher, compared with that in the aged sham-group (Table I; Fig. 1A and B). No change in Trx2 immunoreactivity was observed in the SP 1 day after ischemia-reperfusion (Table I; Fig. 1C). However, as shown in Table I and Fig. 1E, G and I, from 2 days after ischemia-reperfusion, Trx2 immunoreactivity in the SP was markedly decreased in the ischemic CA1 region the adult group, and was almost undetectable, whereas in the aged group, Trx2 immunoreactivity in the SP was significantly increased 1 and 2 days after ischemia-reperfusion, marginally decreased after 4 days after ischemia-reperfusion, was weak 5 days after ischemia-reperfusion (Table I; Fig. 1D, F, H and J) and almost undetectable 7 days after ischemia-reperfusion (Table I).

### Changes in Prx3 immunoreactivity

**Figure 2:**
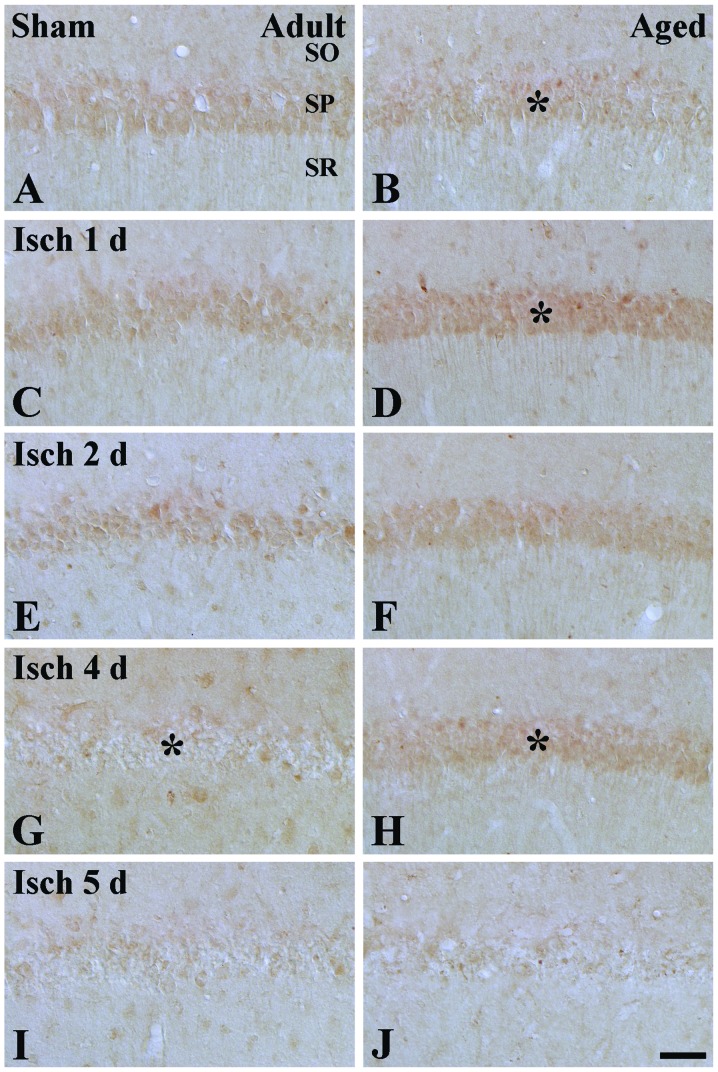
Prx3 immunohistochemistry in the CA1 region of the (A and B) sham-operated and (C-J) ischemia-operated (A, C, E, G and I) adult and (B, D, F, H and J) aged gerbils. In the adult ischemia-group, Prx3 immunoreactivity in the SP (* in G) was decreased from 4 days post-ischemia. In the SP of the aged sham-group, Prx3 immunoreactivity (* in B) was marginally lower, compared with the adult sham-group. However, Prx3 immunoreactivity in the aged ischemia-group was significantly higher (* in D and H), compared with that in the adult ischemia-group. Scale Bar=100 *μ*m. SO, stratum oriens; SR, stratum radiatum; SP, stratum pyramidale; Trx, thioredoxin; Prx, peroxiredoxin; sham, no ischemia-reperfusion.

In the adult sham-group, moderate Prx3 immunoreactivity was detected in the SP of the CA1 region (Table I; Fig. 2A), which was higher than that in the aged sham-group (Fig. 2B). Prx3 immunoreactivity in the SPs of the adult and aged groups was increased 1 and 2 days after ischemia-reperfusion (Table I; Fig. 2C–F). However, as shown in Table I and Fig. 2 G–J, Prx3 immunoreactivity in the SP of the adult and aged groups was weak 4 and 5 days after ischemia-reperfusion, particularly in the adult group, and almost undetectable 7 days after ischemia-reperfusion (Table I).

### Changes in the protein levels of Trx2 and Prx3

**Figure 3:**
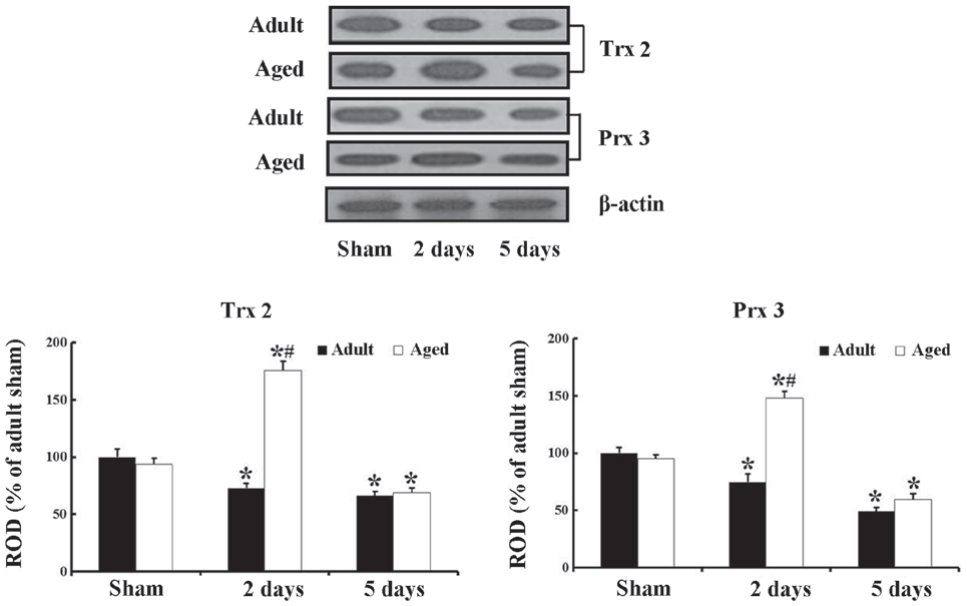
Western blot analysis of Trx2 and Prx3 in the CA1 regions of adult and aged gerbils from the sham- and ischemia-operated-groups. RODs were calculated as percentage values of the immunoblot band (^*^P<0.05, compared with the sham-group; ^#^P<0.05, compared with the adult-group). The data are expressed as the mean ± standard error of the mean. ROD, relative optical density; Trx, thioredoxin; Prx, peroxiredoxin; Sham, no ischemia-reperfusion.

The results of the western blot analysis revealed a similar pattern of changes in the protein levels of Trx2 and Prx3 in the adult and aged hippocampal CA1 region following ischemic damage to those observed in the immunohistochemical data (Fig. 3).

In the adult animals, the protein level of Trx2 was decreased (P=0.0155) from 2 days after ischemia-reperfusion. In the aged sham-group, the protein level of Trx2 was marginally lower, compared with that in the adult sham-group. In the aged ischemia-group, the protein level of Trx2 was significantly increased (P<0.0001) 2 days after ischemia-reperfusion, and a significantly decreased (P=0.0147) 5 days post-ischemia.

The pattern of change in the protein level of Prx3 was similar to that of Trx2. The protein level of Prx3 was also slightly lower, compared with that in the adult sham-group. In the aged ischemia-group, the protein level of Prx3 was also significantly increased (P=0.0001) 2 days post-ischemia, and significantly decreased (P=0.0038) 5 days post-ischemia.

## Discussion

Aging is one of major risk factors affecting neuronal damage in cerebral ischemia (30). In the present study, the transient cerebral ischemia-induced delay of neuronal death was significantly slower in aged gerbils, compared with that in the adult gerbils. This result is in line with other studies and our previous studies, which reported that cerebral ischemia-induced neuronal damage in aged animals is delayed more than in adult animals due to the effects of aging on changes of lysosomes and the caspase-3-dependent apoptotic pathway in the hippocampal CA1 region (27,28,31,32).

In the present study, the protein expression levels of Trx2 and Prx3 in the aged sham-group were marginally lower than those in the adult sham-group. This is the first study, to the best of our knowledge, to demonstrate decreased expression levels of Trx2 and Prx3 in the aged hippocampus. A previous study reported that the protein levels of Prx3 and glutathione-S-transferase ω1 in the human cerebellum is inversely correlated with age (21), and it was suggested that the negative correlation between age and antioxidant proteins was associated with normal aging and oxidative stress. Therefore, it was hypothesized that the decreases of Trx2 and Prx3 in the aged gerbil hippocampus may be associated with increased oxidative stress in aging.

In the present study, the Trx2 immunoreactivity in the SP of the adult ischemia-group was minimal 2 days after ischemia-reperfusion. In the aged animals, Trx2 immunoreactivity in the sham-group was marginally lower than that in the adult sham-group. In the aged ischemia-group, Trx2 immunoreactivity in the SP was significantly higher at 1, 2 and 4 days post-ischemia, compared with that in the adult ischemia-group. At 5 days post-ischemia, Trx2 immunoreactivity was significantly decreased in the SP. Prx3 immunoreactivity in the SP of the adult ischemia-group was significantly decreased from 4 days after ischemia-reperfusion. In the aged animals, Prx3 immunoreactivity in the sham-group was also marginally lower than that in the adult sham-group. Prx3 immunoreactivity in the aged ischemia-group also significantly higher at 1, 2 and 4 days post-ischemia, compared with the adult ischemia-group; however, Prx3 immunoreactivity at 5 days post-ischemia was significantly decreased. The results of the western blot analysis demonstrated similar patterns of change in the protein levels of Trx2 and Prx3 in the adult and aged hippocampal CA1 region following ischemic damage to those observed in the immunohistochemical data. These findings indicated that cerebral ischemia led to different protein expression levels of Trx2 and Prx3 in the hippocampal CA1 region between adult and aged gerbils, and these differences may be associated with increased delay of neuronal death in the aged gerbil hippocampus following transient global cerebral ischemia.

In the present study, ischemia-induced changes in the protein expression levels of Trx2 and Prx3 in the hippocampal CA1 region were examined between adult and aged gerbils. The protein expression levels of Trx2 and Prx3 were markedly decreased in the hippocampal CA1 region of the adult ischemia-group from 2 days after ischemia-reperfusion, and minimal Trx2 and Prx3 immunoreactivity was detected in the adult SP 4 days after ischemia-reperfusion. This result was consistent with those of a previous study, which observed that the immunoreactivities of Trx and Trx mRNA were decreased in the ischemic core region of the rat brain following focal cerebral ischemia (33). By contrast, the present study demonstrated that the immunoreactivities and protein levels of Trx2 and Prx3 in the aged ischemia-group were significantly increased between 1 and 4 days following ischemia-reperfusion, compared with those in the adult ischemia-group. In addition, their levels of expression were almost undetectable in the SP of the aged ischemia-group at 5 days post-ischemia, when delayed neuronal death occurred in the aged ischemia-group. This finding indicated marked expression levels of Trx2 and Prx3 in the neurons of the SP of the aged hippocampal CA1 region, which were maintained significantly longer than those in the adult CA1 region, following transient cerebral ischemia. The present study is also the first, to the best of our knowledge, to demonstrate the changes in the protein expression levels of Trx2 and Prx3 in the aged hippocampus following ischemic insult, therefore, the cause of the difference in the protein expression levels of Trx2 and Prx3 protein expressions between the adult and aged ischemic hippocampal CA1 region is difficult to determine. However, it has is known that age-related changes in mitochondria can lead to the reduction in the production of adenosine triphosphate and excessive oxidative stress, and reductions in antioxidant detoxification mechanisms are associated with increased susceptibility to ischemic damage (34). Therefore, the marked increases in the protein expression levels of Trx2 and Prx3 in the aged ischemia-group may be associated with a compensatory mechanism for increased susceptibility against ischemic damage, although the basal levels of Trx2 and Prx3 in the aged sham-group were marginally lower than those in the adult sham-group.

By contrast, it is widely accepted that the Trx/Prx redox system is closely associated with protective effects against neuronal damage following various insults, including cerebral ischemia (23,24,35). In our previous study, the administration of Prx3 and Prx3/Trx2 into ischemic brains resulted in a substantial neuroprotective effect against ischemic damage by reducing oxidative stress induced by transient ischemia (22).

In conclusion, the results of the present study demonstrated that transient cerebral ischemia led to more marked increase and longer maintenance in the protein expression levels of Trx2 and Prx3 in the hippocampal CA1 region of aged gerbils, compared with adult gerbils. The results indicated that differences in the protein expression levels of Trx2 and Prx3 in the aged gerbil may be associated with the difference in delayed neuronal death in the CA1 region observed between aged and adult gerbils following transient global cerebral ischemia.

## Figures and Tables

**Table I:** Time-dependent levels of Trx2 and Prx3 immunoreactivity in the stratum pyramidale of the hippocampal CA1 region between adult and aged gerbils following transient cerebral ischemia.

Protein	Time following ischemia-reperfusion (days)					
	Sham	1	2	4	5	7
Trx2						
Adult	+	+	−	−	−	−
Aged	+	++	++	++	±	−
Prx3						
Adult	+	+	+	±	±	−
Aged	+	++	++	+	±	−

Trx, thioredoxin; Prx, peroxiredoxin; sham, no ischemia-reperfusion; -, no staining; ±, weakly positive; +, moderate; ++, marked.
